# Sociocognitive Predictors of Condom Use and Intentions Among Adolescents in Three Sub-Saharan Sites

**DOI:** 10.1007/s10508-015-0525-1

**Published:** 2015-04-30

**Authors:** Sander M. Eggers, Leif E. Aarø, Arjan E. R. Bos, Catherine Mathews, Sylvia F. Kaaya, Hans Onya, Hein de Vries

**Affiliations:** 1 Department of Health Promotion, School for Public Health and Primary Care (CAPHRI), Maastricht University, POB 616, 6200 MD Maastricht, The Netherlands; 2 Division of Mental Health, Norwegian Institute of Public Health, Oslo, Norway; 3 Department of Health Promotion and Development, University of Bergen, Bergen, Norway; 4 School of Psychology, Open University, Heerlen, The Netherlands; 5 Health System Research Unit, South African Medical Research Council, Tygerberg, South Africa; 6 School of Public Health and Family Medicine, University of Cape Town, Cape Town, South Africa; 7 Adolescent Health Research Unit, Department of Psychiatry and Mental Health, University of Cape Town, Cape Town, South Africa; 8 Department of Psychiatry and Mental Health, Muhimbili University of Health and Allied Sciences, Dar es Salaam, Tanzania; 9 Department of Public Health Practice and Health Promotion, School of Health Sciences, University of Limpopo-Turfloop Campus, Sovenga, South Africa

**Keywords:** Theory of planned behavior, Condom use, Sub-Saharan Africa, Sexual risk behavior, HIV, AIDS

## Abstract

Many HIV intervention programs in sub-Saharan Africa have applied social cognitive theories such as the theory of planned behavior. However, a recent sub-Saharan African review was unable to show increased effectiveness for theory-based interventions. This study assessed whether the predictive value of attitudes, subjective norms, self-efficacy, and intention was similar to studies in Europe and the U.S., and whether there were differences between three sub-Saharan sites. Longitudinal multigroup structural equation modeling was used to assess whether attitudes, subjective norms, and self-efficacy predicted condom use intentions and condom use (after 6 months) among adolescents in three sites, namely Cape Town (South Africa; *N* = 625), Dar es Salaam (Tanzania; *N* = 271), and Mankweng (South Africa; *N* = 404). Condom use intentions were predicted by subjective norms and self-efficacy in all three sites. Attitudes were not related to intentions in Dar es Salaam and were moderately related to intentions in Cape Town and Mankweng. The proportions of explained variance in intentions and behavior were decent (37–52 and 9–19 %, respectively). Although significant differences in predictive value were found between sites and in comparison to European and U.S. studies, intentions could adequately be explained by attitudes, subjective norms, and self-efficacy. However, the limited proportions of variance in behavior explained by intentions could signify the importance of contextual and environmental factors. Future studies are recommended to use an integrative approach that takes into account both individual and contextual factors, as well as social and environmental differences.

## Introduction

Promoting condom use has received increasing attention around the world in the last few decades due to a steep rise in HIV prevalence. Although recent estimates point to a decline in HIV/AIDS-related mortality and incidence, HIV is still a huge problem in sub-Saharan Africa with prevalence rates ranging from 2 % (Ghana) to 26 % (Swaziland) (UNAIDS, 2010). In order to combat the epidemic, it is essential that HIV interventions implemented in sub-Saharan Africa use theoretical frameworks that are relevant and effective. One of the most prominent frameworks for explaining the link between cognition and behavior is the theory of planned behavior (TPB) (Ajzen, 1991). Social science scholars have used this theory for a wide range of behaviours, including condom use, since the early 1990s (Armitage & Conner, 2001; Godin & Kok, 1996). A study by Kirby, Laris, and Rolleri (2007) showed that, worldwide, 29 % of the sex education programs included in their review used the TPB or its predecessor, the Theory of Reasoned Action (TRA), in the development or evaluation phase. For sub-Saharan Africa specifically, Michielsen, Chersich, Temmerman, Dooms, and Van Rossem (2012) reported that 50 % of the HIV interventions included in their review, used sociocognitive theories to inform content and 24 % used the TPB or the TRA. Interestingly, their study was unable to provide evidence of increased effectiveness for theory-based HIV-prevention programs compared to non-theory-based programs. This implies that sociocognitive theories, such as the TPB, may have less explanatory power in sub-Saharan Africa, or that their implementation in practice might be flawed. This study addressed the former line of reasoning by testing whether attitudes, subjective norms, self-efficacy, and intention predict condom use in three different sub-Saharan sites, namely Cape Town (South Africa), Dar es Salaam (Tanzania), and Mankweng (South Africa).

### The Theory of Planned Behavior

The TPB postulates that intention is the main determinant of behavior (Ajzen, 1991). Three proximal factors are hypothesized to predict intention, namely attitudes, subjective norms, and perceived behavioral control (PBC). Attitudes can be defined as positive or negative evaluations of executing the desired behavior (e.g., “Using a condom is pleasant”). Subjective norms are beliefs about what significant others, such as friends and family, think the individual should do (e.g., My family thinks I should use a condom) and PBC is the expected ease or difficulty to perform the desired behavior (e.g., “Using a condom when I am drunk is [easy-difficult]”). According to Ajzen (2012), PBC is conceptually similar to self-efficacy, a term introduced by Bandura (1977). Others, however, have argued that they are conceptually distinct (Trafimow, Sheeran, Conner, & Finlay, 2002). Since previous studies have shown that self-efficacy explains most of the variance in intentions in comparison to PBC (Armitage & Conner, 2001; Manstead & van Eekelen, 1998; Terry & O’Leary, 1995; White, Terry, & Hogg, 1994), only self-efficacy was assessed in the present study.

From a meta-analysis by Albarracin, Johnson, Fishbein, and Muellerleile (2001) of 96 condom use studies, of which 82 took place in Europe or the U.S. and an additional 9 in Australia, it was calculated that, on average, attitudes, subjective norms and PBC accounted for 53 % of the variance in intentions to use a condom. In addition, their meta-analysis showed that attitudes was one of the most important determinants of intention (*r* = .58, *β* = .47), followed by PBC (*r* = .45, *β* = .20) and subjective norms (*r* = .39, *β* = .21). These TPB factors, including intentions, are known to explain roughly a third of the behavioral variance, depending on the behavior in question (Armitage & Conner 2001; Godin & Kok 1996; McEachan, Conner, Taylor, & Lawton, 2011).

### Application in sub-Saharan Africa

Previous research has shown that the overall amount of explained variance, as well as the individual contribution of specific TPB factors, is potentially lower in sub-Saharan Africa than in Europe or the U.S. (see Table 1). A study by Bryan, Kagee, and Broaddus (2006), for example, assessed whether TPB variables prospectively predicted the intention to use condoms (assessed four months after baseline) among adolescents in Cape Town, South Africa. They found that subjective norms were the most predictive construct, followed by self-efficacy and attitudes. Together, these variables accounted for only 22 % of the variance in intentions. Likewise, a study by Boer and Mashamba (2007) assessed correlates of condom use cross-sectionally among adolescents in Venda, South Africa, and found that TPB variables accounted for a third of the variance in intentions to use a condom (38 % for males and 22 % for females) and that subjective norms were the most predictive construct among males. Furthermore, Lugoe and Rise (1999) found that PBC was the most predictive construct among adolescents in Tanzania, and that PBC, subjective norms, and attitudes accounted for 42 % of the variance in intentions to use a condom. In sum, only 1 out of 12 sub-Saharan studies was able to find estimates of explained variance that exceeded 43 % (see Table 1). These findings suggest that the predictive accuracy of the TPB is relatively low in sub-Saharan Africa.

**Table 1:** Overview of sub-Saharan African studies that have assessed the TPB in relation to condom use

Authors	Design	R^2^	Attitudes		Norms		PBC	
			*r*	*β*	*r*	*β*	*r*	*β*
Boer and Mashamba (2005)	CR	.17	.05	−.02	.43	.39	.22	.02
Boer and Mashamba (2007): Males	CR	.38	.31	.25	.56	.42	.40	.14
Boer and Mashamba (2007): Females	CR	.22	.40	.39	.27	.17	.08	.04
Bosompra (2001)	CR	–	.32	.15	.54	.45	–	–
Bryan et al. (2006)^a^	PR	.22	.22	.12	.40	.29	.34	.24
Fekadu and Kraft (2001)	CR	.27	.33	.20	.46	.33	.24	.18
Giles, Liddell, and Bydawell (2005)^a^	PR	.67	.57	.03	.62	.35	.53	.09
Heeren, Jemmott, Mandeya, and Tyler (2007)^a^	CR	.35	–	.27	–	.32	–	.40
Jemmott et al. (2007)	CR	.37	.45	.16	.29	.05	.57	.46
Molla, Nordrehaug Åstrom, and Brehane (2007)	PR	.36	.66	.41	.64	.31	.52	.10
Schaalma et al. (2009)^a^	CR	–	–	.12	–	.27	–	.41
Lugoe and Rise (1999)	CR	.42	.37	.11	.42	.22	.59	.48
Protogerou, Flisher, Wild, and Aarø (2013): Sexually active	CR	.43	.56	.40	.50	.30	.31	.28
Protogerou et al. (2013): Sexually inactive	CR	.31	–	.37	–	.00	–	.16

*CR* measured intention and predictors cross-sectionally, *PR* measured intention and predictors prospectively

^a^Self-efficacy was measured instead of PBC

### The Present Study

In the past, the applicability of Western-based sociocognitive models in other cultural settings has been questioned (Airhihenbuwa & Obregon, 2000; Diaz, 1999) and the results presented in Table 1 indicate that the overall amount of explanatory power is indeed somewhat lower in sub-Saharan studies. Intuitively, it seems plausible to assume that motivational processes may differ between cultures or geographical regions. The influence of social norms, for example, could be stronger in sub-Saharan Africa because traditional African society is more anchored in family ties and collectivism (Afrocentric Alliance, 2001; Airhihenbuwa & Obregon, 2000; Oyserman, Coon, & Kemmelmeier, 2002). The question arises if these differences with Western studies are uniform across sub-Saharan Africa. Previous studies have shown large differences between sub-Saharan regions in STI prevalence and condom use which could partly be explained by differences in marital status, male circumcision rates, economic factors, rate of partner change, and other factors (Auvert et al., 2001; Bongaarts, 2007; Ferry et al., 2001; UNAIDS, 2010; Weiss et al., 2001). To our knowledge, no previous study has directly assessed whether there are significant differences in motivational processes between regions which subsequently may explain differences in condom use and HIV prevalence.

In this study, the predictive value of the TPB was assessed in three different sub-Saharan sites. An important difference between these sites is their demographic profile (City Council Dar es Salaam, 2004; National Bureau of Statistics, 2011; South Africa Census, 2011). Cape Town, for example, is a highly developed multicultural city with roughly 3.7 million inhabitants and dates back to the seventeenth century. It is situated in the Western Cape province, which has the lowest HIV prevalence rate of the South African provinces (5.3 % for the age group: 15–49) (Shisana et al., 2009). Dar es Salaam is slightly smaller than Cape Town (3 million inhabitants) and is more uniform racially. It is also the third fastest growing city in Africa (after Bamako and Lagos) and has an infrastructure and educational system that is upgraded continuously. The prevalence of HIV in Dar es Salaam is estimated to be 7.0 % (age 15–49) (Tanzania Commission for AIDS, 2013). In comparison to these two sites, Mankweng is much smaller, both in size and population (20,000) and is more rural. It is situated in the South African province of Limpopo. HIV prevalence in Limpopo is estimated to be 13.7 % (age 15–49) (Shisana et al., 2009).

To assess whether attitude, subjective norms, self-efficacy, and intention predicted condom use similarly across these three sites, measurement invariance between the sites was assessed to rule out the possibility that differences in coefficients were caused by different interpretations of the items. Second, the proposed pathways of TPB will be tested in a multigroup structural equation model that predicted condom use at follow-up, 6 months after baseline. Through this study, insight will be gained in the cross-regional applicability of sociocognitive factors such as attitudes, subjective norms, self-efficacy, and intention and their prospective predictive power.

## Method

### Participants

All participants (*N* = 1166) were sexually active students enrolled in 38 primary or high schools in either Cape Town (12 schools, *N* = 564), Dar es Salaam (12 schools, *N* = 215) or Mankweng (15 schools, *N* = 387). The schools in each site were selected to be representative of the public schools in each corresponding geographic area. The students’ age ranged from 11 to 18 years and three quarters of the sample were male (see Table 2). Data collection was part of a randomized controlled trial (RCT) of a school-based HIV prevention program (i.e., SATZ program; *N* = 12,139) which took place during 2004 and 2005. The program was based on TPB and aimed at increasing pro-condom attitudes, self-efficacy beliefs, and positive subjective norms of students and is described in more detail by Aarø et al. (2006) and Mathews et al. (2012).

**Table 2:** Sample characteristics: Observed means and percentages

	Total (*N* = 1166)		Cape Town (*N* = 564)		Dar es Salaam (*N* = 215)		Mankweng (*N* = 387)		Difference CT versus Dar	Difference CT versus Mank	DifferenceDar versus Mank
	*M*	*SE*	*M*	*SE*	*M*	*SE*	*M*	*SE*			
Age	14.1	1.5	14.2	0.30	13.4	0.20	14.3	0.17	*F*(1, 22) = 5.5*	*F*(1, 26) = 0.2	*F*(1, 25) = 14.7**
Male % (*N*)	73 %	(852)	68 %	(387)	78 %	(169)	76 %	(296)	*χ*²(22) = 7.6*	*χ*²(26) = 7.0	*χ*²(25) = 0.4
SES	2.9	1.4	3.3	0.21	2.5	0.19	2.7	0.16	*F*(1, 22) = 7.9*	*F*(1, 26) = 4.3*	*F*(1, 25) = 1.0
Attitudes	3.86	0.85	3.96	0.04	3.63	0.09	3.85	0.07	*F*(1, 22) = 11.1**	*F*(1, 26) = 1.6	*F*(1, 25) = 3.8
Subjective norms	3.84	0.93	3.98	0.07	3.41	0.09	3.88	0.06	*F*(1, 22) = 25.1***	*F*(1, 26) = 1.4	*F*(1, 25) = 18.2***
Self-efficacy	3.72	0.85	3.89	0.04	3.29	0.07	3.72	0.05	*F*(1, 22) = 48.1***	*F*(1, 26) = 6.4*	*F*(1, 25) = 24.4***
Intention	3.98	1.12	4.07	0.05	3.58	0.10	4.06	0.08	*F*(1, 22) = 19.9***	*F*(1, 26) = 0.1	*F*(1, 25) = 14.9**
Sexually active (*N*)^a^	28 %	(3417)	34 %	(1840)	16 %	(572)	34 %	(1005)	*χ*²(27) = 359.0***	*χ*²(29) = 0.3	*χ*²(29) = 304.7***
Condom use % T1 (*N*)	30 %	(352)	33 %	(190)	14 %	(29)	34 %	(133)	*χ²*(22) = 31.4***	*χ*²(26) = 0.1	*χ*²(25) = 30.6***
Condom use % T2 (*N*)	37 %	(434)	41 %	(232)	22 %	(48)	40 %	(154)	*χ*²(22) = 23.9**	*χ*²(26) = 0.2	*χ*²(25) = 18.9**

All confidence intervals were corrected for the cluster effect of students within schools

*CT* Cape Town, *Dar* Dar es Salaam, *Mank* Mankweng

*p < .05, **p < .01, ***p < .001

^a^Proportions based on full sample (*N* = 16,524)

### Procedure

Data collection took place during classroom sessions with a member of the research team present. No educators were present during these sessions. The questionnaire was administered in English, Afrikaans and Xhosa in Cape Town, in Kiswahili in Dar es Salaam and in Sepedi in Mankweng. In Dar es Salaam and Mankweng, the ID-coded paper questionnaires were put in sealed envelopes after completion and collected by a member of the data collection team. In Cape Town, electronic questionnaires on hand-held computers (personal digital assistant, PDA) were used (Seebregts et al., 2009). The data collection team used a list, prepared in advance, with all ID-numbers and participant names to guarantee correct pairing of questionnaires at follow-up. Names of students were not recorded in questionnaires or data files and only members of the data collection team had access to the list to ensure anonymity. Participation was voluntary and informed consent forms were signed before filling out the questionnaire. Parents received letters describing the study and could complete a declination form to prevent participation (only 0.01 % of the parents declined). Ethical clearance was provided by the Western Norway Regional Committee for Medical Research Ethics, the Ethics Committee of the Faculty of Health Sciences, University of Limpopo, the Senate Research and Publications Committee of the Muhimbili University of Health and Allied Sciences in Dar es Salaam, and the Research Ethics Committee of the University of Cape Town. For the present analyses, only data were used from students who were sexually active at baseline and took part in both baseline and follow-up data collection (6 months after baseline).

### Measures

The self-report questionnaire consisted of items that had been used in previous HIV-prevention studies in African settings (Flisher, Ziervogel, Chalton, Leger, & Robertson, 1993; Kirby et al., 2004; Klepp, Ndeki, Leshabari, Hannan, & Lyimo, 1997; Vergnani, Flisher, & Blignaut, 2002; WHO, 2004) and was revised based on qualitative research with members of the target group in each site (*N* = 50 per site). Items were subsequently translated and back-translated and the resulting instrument consisted of 155 items that addressed condom use and delaying sexual debut. Test–retest studies showed that the internal consistency and test–retest correlations of the scales were adequate (Mukoma et al., 2009). For the purpose of this study, only those items were used that specifically addressed attitudes, subjective norms, self-efficacy, intentions to use condoms, condom use at last intercourse, gender, socioeconomic status (SES), and age.

#### Attitudes

Five items assessed whether the participant felt that it was “ok” to carry condoms, whether it was “ok” to use condoms, whether it was “ok” to suggest condom use to a partner, and whether they felt that using a condom was showing responsibility for themselves, and for their partner. Based on the qualitative research findings, the term “ok” was used instead of more conventional formulations. First, because it was common language for our target group; and second, because it had a uniform interpretation between sites. Response options ranged from 1 (*strongly disagree*) to 5 (*strongly agree*). Cronbach’s alpha = 0.74 for Cape Town, 0.83 for Dar es Salaam, and 0.71 for Mankweng.

#### Subjective Norms

Four items assessed whether their parents thought that the participant should carry a condom, whether friends thought that the participant should carry a condom, whether parents thought that the participant should actually use a condom, and whether friends thought that the participant should actually use a condom. The response options ranged from 1 (*strongly disagree*) to 5 (*strongly agree*). Cronbach’s alpha = 0.75 for Cape Town, 0.84 for Dar es Salaam, and 0.76 for Mankweng.

#### Self-Efficacy

Eight items assessed how able the participant felt to negotiate condom use with a potential partner, to refuse sex if no condom was available, to use condoms consistently with a steady partner, to use a condom when intoxicated, to apply a condom correctly, to use condoms consistently no matter what the circumstance, to apply a condom without spoiling the mood, and to discuss condom use in general. Response options ranged from 1 (*strongly disagree*) to 5 (*strongly agree*). Cronbach’s alpha = 0.81 for Cape Town, 0.90 for Dar es Salaam, and 0.78 for Mankweng.

#### Intention

One item assessed whether participants intended to use a condom: “I plan to use a condom when I have sexual intercourse”. Participants could reply on a five-point Likert scale ranging from 1 (*strongly disagree*) to 5 (*strongly agree*).

#### Socioeconomic Status

SES was assessed by using an adapted Family Affluence Scale (Currie et al., 2008), that asked participants which of the following assets they had access to at their homes: a car, a television, electricity, tap water, or a bicycle. Participants could answer with *yes* (1) or *no* (0) for each item. Results were subsequently summed to create a family affluence scale. This approach was considered to be more appropriate than using the parents’ educational level or income, since many of the students did not live with their biological parents and because of the difficulty to interpret differences in educational and income levels between the three sites.

#### Sexual Activity

Participants were asked whether they ever had vaginal or anal sex and could respond with *yes* (1) or *no* (0).

#### Relationship Status

Participants were asked whether they ever had a boy or girlfriend and could respond with *yes* (1) or *no* (0).

#### Condom Use

Participants were asked whether they had used a condom during their last sexual intercourse and could respond with *yes* (1) or *no* (0).

### Statistical Analysis

SPSS 19 was used to enter the data and to describe sample characteristics. Measurement invariance was assessed with Mplus Version 6 by following the procedures outlined by Van der Schoot, Lugtig, and Hox (2012). In their study, they described a four-step approach to assess measurement invariance: First, metric invariance was tested by allowing the intercepts to differ between sites and by keeping the factor loadings constant. Second, the intercepts were constrained to be equal across sites but the factor loadings were allowed to differ to assess whether the meaning of the levels of the underlying items differed per site. Third, scalar invariance was tested by constraining the intercepts and factor loadings to be equal across sites. In the fourth and final step, full uniqueness measurement invariance was tested by also constraining the residual variances to be equal across sites.

Structural equation modeling in Mplus was used to regress condom use at follow-up on intention and demographics (age, gender, SES) at baseline. Subsequently, intention was regressed on attitudes, subjective norms, and self-efficacy. Additionally, according to several recommendations (Ajzen, 2002; Ouellette & Wood, 1998; Rhodes & Courneya, 2003), the added predictive value of previous behavior was assessed by including it as a predictor of the cognitive factors, which implies that cognitive factors should mediate the effect of previous behavior on intention and future behavior. Modification indices were checked to improve model fit if necessary (MI > 25), but no modifications were needed. The Delta method was used to calculate indirect effects and we adjusted for the nested structure of students within schools by using the cluster option available in Mplus (Muthén & Muthén, 2010). Neglecting this nested structure can lead to substantial underestimation of standard errors. Chi square tests alone are insufficient to assess model fit (Hu & Bentler, 1995). This study therefore also used the comparative fit index (CFI), the Tucker-Lewis Index (TLI), and root mean square error of approximation (RMSEA) to determine model fit. For the CFI and TLI, values over 0.95 indicate good fit and for the RMSEA, which controls for sample size, values below 0.06 indicate good fit (Hu & Bentler, 1999) and values below 0.08 indicate adequate fit (Van der Schoot et al., 2012). Potential differences in effect sizes were assessed with the Wald test and differences in explained variance were assessed by applying Fischer’s *z* transformations (Meng, Rosenthal, & Rubin, 1992).

All items that were assumed to reflect latent factors were defined as categorical and the standard estimator, weighted least squares mean and variance adjusted (WLSMV), was used to calculate coefficients. Previous research has shown that this estimator performs well under a wide range of conditions (Flora & Curran, 2004). The presented coefficients are unstandardized since all predictors of interest (attitudes, subjective norms, self-efficacy, and intention) used identical five point scales. Effects were considered significant when *p* < .05.

## Results

### Sample Characteristics

On average, the students were 14.1 years of age (*SD* = 1.57) and 74 % were male. In general, students had access to an average of 2.9 (out of five) assets (*SD* = 1.45) at their homes. The sites differed significantly in terms of demographic characteristics, with Dar es Salaam having younger participants, more males, and fewer household assets (see Table 2). At baseline, the overall reported prevalence of condom use during last sexual intercourse was low (30 %). As can be seen in Table 2, condom use rates were nearly equal in Cape Town (33 %) and Mankweng (34 %). Dar es Salaam, however, had a significantly lower condom use rate of 14 %. Baseline results from the full available sample (including those who never had sex; *N* = 16,524) showed that Dar es Salaam had a significantly lower proportion of students (16 %) who were sexually active (34 % for Cape Town and Mankweng) and a significantly lower proportion of students that ever had a boy or girlfriend (33 % in Dar es Salaam compared to 82 % in Cape Town, *p* < .01; and 48 % in Mankweng, *p* < .01). After 6 months, reported condom use rates were slightly higher in all three sites (Cape Town: 41 %; Mankweng: 40 %), with Dar es Salaam still reporting the lowest rate of condom use (22 %). Concerning cognitive factors, Dar es Salaam scored significantly lower on subjective norms, self-efficacy, and intentions to use a condom (see Table 2).

### Factor Structure

First, to assess measurement invariance, the factor structure was tested for each site separately. The items assumed to reflect latent constructs had similar, but not identical, factor loadings in all three sites. Confirmatory factor analysis indicated that all loadings were higher than 0.40 (*p* < .01) and therefore met the criterion proposed by Stevens (1992). Model fit was sufficient for each site and differences between the three sites were small (see Table 3). Next, four invariance models were tested (measurement invariance, intercept only invariance, metric invariance, and full uniqueness invariance), following the procedures outlined by Van der Schoot et al. (2012). All four models performed adequately (CFI > .90; TLI > .90; RMSEA < .08), which implies that the factor structure was similar across sites and that measurement equivalence was sufficiently present for testing multigroup differences.

**Table 3:** Model fit estimates

	χ^2^	df	CFI	TLI	RMSEA
1. Configural invariance					
Cape Town (*N* = 564)	233	116	.97	.97	.03
Dar es Salaam (*N* = 215)	229	116	.98	.98	.02
Mankweng (*N* = 387)	205	116	.97	.96	.02
2. Metric invariance	1125	382	.95	.95	.06
3. Intercept only invariance	1309	484	.94	.95	.06
4. Scalar invariance	1456	484	.93	.94	.07
5. Full uniqueness invariance	1625	501	.92	.94	.08

All χ^2^ estimates have *p* < .001

### Correlates of Condom Use

Attitudes, subjective norms, and self-efficacy were weakly to moderately correlated with condom use at baseline and at follow-up (see Table 4). In addition, correlations between intentions at baseline and condom use at follow-up were markedly low in Mankweng (*r* = .02), Cape Town (*r* = .26) and Dar es Salaam (*r* = .23). Also notable was the negative correlation between gender and TPB factors in Dar es Salaam and Mankweng, implying that girls were less positive about condom use, perceived less social norms, and less self-efficacy to use a condom in comparison to boys.

**Table 4:** Estimated correlations between factors

	1	2	3	4	5	6	7	8
Cape town								
1. Age								
2. Gender	.10							
3. SES	−.27**	−.19**						
4. Attitudes	−.01	.01	.02					
5. Subjective norms	−.14*	.03	.16*	.68***				
6. Self-efficacy	−.07	.10*	.08	.64***	.72***			
7. Intention	−.02	.02	.05	.58***	.60***	.57***		
8. Condom use T1	.05	−.02	.02	.08*	−.05	.07	.07	
9. Condom use T2	−.05	.06	.16*	.27***	.27***	.27***	.26***	.31***
Dar es Salaam								
1. Age								
2. Gender	−.08							
3. SES	−.21**	.11						
4. Attitudes	.20***	−.14**	−.26***					
5. Subjective norms	.18***	−.23**	−.16**	.60***				
6. Self-efficacy	.20***	−.23**	−.14**	.66***	.76***			
7. Intention	.19**	−.24**	−.09	.53***	.70***	.68***		
8. Condom use T1	.29***	−.02	−.03	.15	.20	.16**	.20*	
9. Condom use T2	.28***	.19*	−.07	.12	.05	.14*	.23***	.51***
Mankweng								
1. Age								
2. Gender	.10							
3. SES	−.16***	−.14*						
4. Attitudes	.07	−.17**	.01					
5. Subjective norms	.10*	−.16**	.02	.52***				
6. Self-efficacy	.05	−.02	−.03	.52***	.66***			
7. Intention	−.03	.05	.18**	.46***	.51***	.55***		
8. Condom use T1	.28***	−.23*	.07	.22**	.34***	.28***	.15	
9. Condom use T2	.24**	−.07	−.01	.17**	.18*	.20**	.02	.82***

All correlations were estimated in Mplus and corrected for the cluster effect

*T1*  baseline,* T2* 6 month follow-up

* *p* < .05, ** *p* < .01, *** *p* < .001

### The TPB Model

**Fig. 1:**
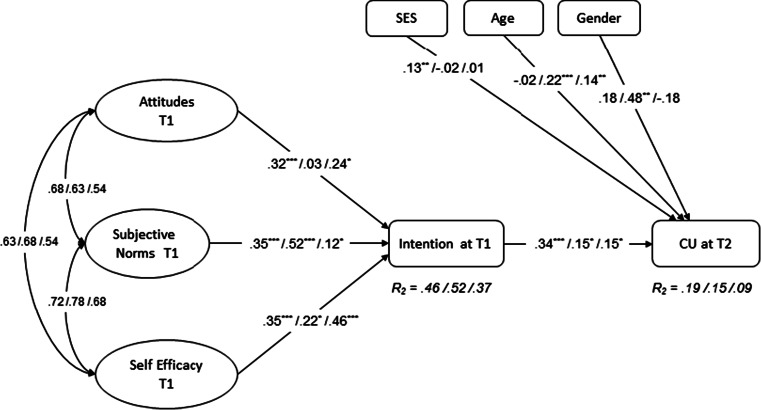
TPB model without baseline condom use (*N* = 1166; χ^2^ = 1020; df = 727; *p* < .001; CFI = .97; TLI = .98; RMSEA = .031): *Double headed arrows* are correlations and *single headed arrows* unstandardized regression coefficients. Ellipses are latent factors and rectangles represent single-item indicators. Coefficients are presented in the following order: Cape Town/Dar es Salaam/Mankweng

The basic assumptions of the TPB were tested by regressing reported condom use at follow-up on baseline intention while adjusting for SES, age, and gender. Baseline intention was subsequently regressed on baseline attitudes, subjective norms, and self-efficacy (Fig. 1; CFI = .97; TLI = .98; RMSEA = .031). The results showed that intention was an important determinant of condom use at follow-up for all three sites, with Cape Town having a significantly larger coefficient than the other two sites (see Table 5). The influence of demographic factors varied per site. SES was significantly associated with condom use in Cape Town (*B* = 0.13), but not in the other two sites. Being older significantly predicted condom use in Dar es Salaam (*B* = 0.22) and Mankweng (*B* = 0.14) and being female was significantly associated with condom use in Dar es Salaam (*B* = 0.48). The proportion of explained behavioral variance by intention alone was very low for Mankweng (5 %) and Dar es Salaam (5 %; *p* > .05) and somewhat higher for Cape Town (18 %; *p* < .05).

**Table 5:** Significance of the differences in coefficients between Cape Town, Dar es Salaam and Mankweng

	CT versus Dar		CT versus Mank		Dar versus Mank	
	Wald	*p* value	Wald	*p* value	Wald	*p* value
Previous behavior excluded						
Attitudes-intention	11.91	<.001	0.50	ns	3.44	.064
Subjective norms-intention	1.98	ns	4.33	.038	22.58	<.001
Self-efficacy-intention	0.90	ns	0.82	ns	2.81	.094
Intention-condom use	4.17	.041	4.79	.029	0.01	ns
SES-condom use	5.34	.021	3.61	.057	0.27	ns
Age-condom use	12.07	<.001	6.90	.009	0.89	ns
Gender-condom use	1.96	ns	3.48	.062	8.10	.004
Previous behavior included						
Attitudes-intention	12.45	<.001	0.38	ns	3.70	.054
Subjective norms-intention	1.23	ns	5.06	.024	16.78	<.001
Self-efficacy-intention	2.40	ns	1.31	ns	5.56	.018
Intention-condom use	9.83	.002	27.67	<.001	0.51	ns
SES-condom use	5.38	.020	7.02	.008	0.22	ns
Age-condom use	6.41	.011	1.01	ns	1.45	ns
Gender-condom use	3.30	.069	0.32	ns	5.02	.025
Previous behavior-condom use	0.69	ns	21.56	<.001	5.37	.021

*CT* Cape Town, *Dar* Dar es Salaam, *Mank* Mankweng

In Dar es Salaam, attitudes were not associated with the intention to use a condom. In the other two sites, attitudes contributed significantly in the prediction of intentions to use a condom (Cape Town: *B* = 0.32; Mankweng: *B* = 0.24). Subjective norms showed moderate to large associations with intention in both Cape Town (*B* = 0.35) and Dar es Salaam (*B* = 0.52), but was of significantly less importance in Mankweng (*B* = 0.12). Self-efficacy had a moderate association with intention in all three sites, with no significant differences between sites (see Table 5). The proportion of explained variance was significantly higher in Cape Town (46 %, *Z* = 2.70) and Dar es Salaam (52 %; *Z* = 4.68) in comparison to Mankweng (37 %; both *ps* < .05).

**Fig. 2:**
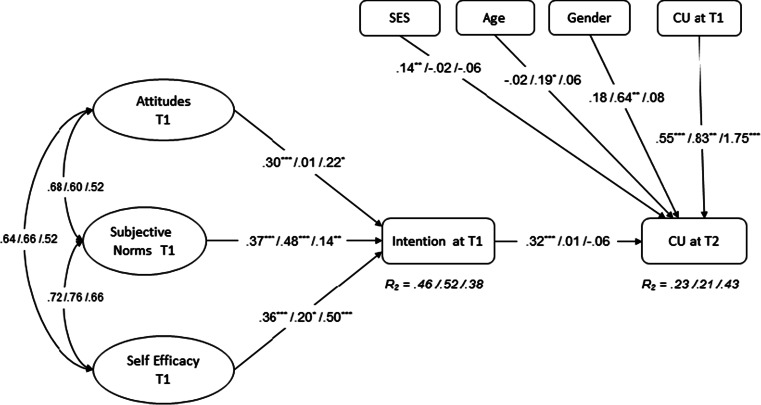
TPB model with baseline condom use (*N* = 1143; χ^2^ = 1058; df = 781; *p* < .001; CFI = .97; TLI = .97; RMSEA = .031): *Double headed arrows* are correlations and *single headed arrows* unstandardized regression coefficients. Ellipses are latent factors and rectangles represent single-item indicators. Coefficients are presented in the following order: Cape Town/Dar es Salaam/Mankweng

Three alternative models were tested that did not result in a better fit. First, since previous behavior is an important predictor of future behavior, baseline condom use was included in the model as an exogenous factor (Fig. 2; CFI = .97; TLI = .97; RMSEA = .031). The results showed that baseline condom use was associated with condom use at follow-up in all three sites. In Mankweng, however, baseline condom use had a significantly larger influence than in the other two sites. In addition, none of the three demographic variables were associated with condom use after 6 months in Mankweng and neither was intention. Intention and SES were only significantly associated with condom use in Cape Town (*B* = 0.32 and 0.14, respectively) and being female was significantly associated with condom use in Dar es Salaam (*B* = 0.64). With the inclusion of baseline condom use, the proportion of explained behavioral variance increased significantly in Dar es Salaam (ΔR^2^ = 6 %), and Mankweng (ΔR^2^ = 34 %), but not in Cape Town (ΔR^2^ = 4 %; *p* > .05). No significant changes occurred for the effect sizes of attitudes, subjective norms, and self-efficacy on intention.

A second alternative model was tested which included direct effects of previous behavior on cognitive factors, since other researchers have suggested that the cognitive factors should mediate the effect of previous behavior (Ajzen, 2002; Ouelette & Wood, 1998; Rhodes & Courneya, 2003). However, when applying this method, it was found to have a detrimental effect on model fit (CFI = .55, TLI = .57, RMSEA = .12). Hence, there was only support for a direct effect of previous behavior which was not mediated by the cognitive factors.

Third, for behaviors that are not fully under volitional control, Fishbein and Ajzen (2010) hypothesized, besides an indirect effect, a direct effect from self-efficacy to behavior. In the current study, the direct effect of self-efficacy was not significant in Dar es Salaam and Mankweng. In Cape Town, however, there was a moderate link between self-efficacy and condom use at follow-up (*B* = 0.32, *p* < .01). When included, the direct effect of intention decreased considerably (*B* = 0.11; *p* < .05). No other significant changes were observed.

### Indirect Effects of Attitudes, Subjective Norms, and Self-Efficacy on Behavior

Significant mediation by intention (see Fig. 1) was found for subjective norms in all three sites (Cape Town: *ab* = 0.12; Dar es Salaam: *ab* = 0.08; Mankweng: *ab* = 0.02; all *ps* < .05). Effects of self-efficacy on condom use were only mediated by intention in Cape Town (*ab* = 0.12; *p* < .05) and Mankweng (*ab* = 0.07; *p* < .05), and attitudes were only mediated by intention in Cape Town (*ab* = 0.11; *p* < .05).

## Discussion

The results illustrated that attitudes, subjective norms, self-efficacy, and intention were able to predict condom use prospectively across the three sites. The data suggested that the conceptual identity (i.e., meaning, interpretation) of the separate items and their underlying factors were similar across the sites. However, their relevance for explaining behavior and intention varied across sites. In Cape Town, all three motivational factors were significantly associated with intention and indirectly with behavior and the proportion of variance in intention that was accounted for by the model (46 %) was up to par with most European and U.S. studies (Albarracin et al., 2001; Armitage & Conner, 2001). In Dar es Salaam, the proportion of explained variance was similar (52 %), but there was no direct association between attitude and intention and only a small association between intention and behavior. In Mankweng, the proportion of variance in intention that was accounted for (37 %) was significantly smaller than in the other two sites and only a small association between intention and behavior was found. When taking into account the age of these students and their puberty-related shifting norms and identity profile, intention-behavior associations of 0.15 to 0.33 over a time period of 6 months can be regarded as quite decent.

It is interesting that the association between intention and behavior was significantly stronger in Cape Town when compared to the other two sites. For condom use, translating the intention to use a condom into actual behavior requires at least three steps. First, it requires negotiation with a partner at a highly emotional, and sometimes impulsive, moment. Next, if the negotiation is successful, some knowledge about how to unpack and correctly apply a condom is required. Third, one needs sufficient foresight to know when to buy and carry condoms to be able to use them (Van Empelen & Kok, 2008). Explanations for the differential effects of intention could be attributed to differences in any of these steps. With regard to the first step, the results showed that girls reported lower attitudes, subjective norms, and self-efficacy scores in comparison to boys in both Dar es Salaam and Mankweng. This may imply that traditional gender roles, in which the male is supposed to take the initiative and is regarded as “responsible” for safe sex practices such as condom buying and negotiating, are more prevalent in these sites. Such gender roles are known to inhibit safe sex practices and may partially explain the lack of congruence between intentions and behavior (Pulerwitz, Amaro, Jong, Gortmaker, & Rudd, 2002; Varga, 2003). With regard to the first and second step, the self-efficacy scores showed that students in Cape Town felt more able to negotiate, discuss, and apply condoms than students from Mankweng and Dar es Salaam. This difference in self-efficacy could potentially have led to a stronger association between intention and behavior. Additionally, students in Cape Town reported higher family affluence scores than their counterparts in the other two sites. If students in Cape Town were more affluent, this may imply that they were more informed about the importance of condom use or less susceptible to the economic benefits of transactional sex, which is common in sub-Saharan Africa (Luke, 2005; Meekers & Calves, 1997). Finally, with regard to the third step, access to condoms may differ considerably across sites. Even though all three sites are obliged by the government to provide free access to condoms through public health clinics, research has shown that stigmatization by staff members and a general lack of privacy are significant barriers for adolescents who intend to visit a health clinic in search for condoms (MacPhail & Campbell, 2001; Rasch, Silberschmidt, McHumvu, & Mmary, 2000). Unfortunately, no up-to-date empirical data on the perceived availability of condoms in the three sites of interest exist. Future research should therefore identify potential moderators of the intention-behavior association, such as perceived access to condoms, which may explain geographical differences in condom use.

Another interesting finding was the relatively small effect size of attitudes in comparison to studies from Europe and the U.S. (Albarracin et al., 2001), particularly in Dar es Salaam. One potential explanation is the relative inexperience of most students with the subject, since the overall proportion of sexually active students was only 34 % for Cape Town and Mankweng, and 16 % in Dar es Salaam. Even though this study used a subsample of sexually active students, the relatively young age of the students and the associated inexperience may imply that personal cognitions such as “attitudes towards condoms” are still developing along with the experience with sex and condoms (Montemayo, Adams, & Gullota, 1990). In addition, the participants in Dar es Salaam were significantly younger than those in Cape Town and Mankweng, which further strengthens the argument that inexperience with sex and condom use may explain the lack of effect in that particular site. Another explanation, previously suggested by Aarø, Schaalma, and Åstrøm (2008), might be that some studies show that individuals in sub-Saharan Africa are more collectivistic than those in Western Europe or the U.S. (Afrocentric Alliance, 2001; Airhihenbuwa & Obregon, 2000; Eaton & Louw, 2000; Oyserman et al., 2002). In such a context, individualistic thoughts and attitudes towards behavior might be less influential than social norms and values. This explanation is somewhat supported by our results, since subjective norms were strongly associated with intention in two of the three sites. Moderate to large effect sizes of subjective norms with intention are also confirmed by other studies in Africa (Boer & Mashamba, 2007; Bryan et al., 2006; Fekadu & Kraft, 2002), but are less common in studies from Europe and the U.S. (Albarracin et al., 2001; Armitage & Conner, 2001; Godin & Kok, 1996; Sheeran, Abraham, & Orbell, 1999).

Although the results suggest potential differences in explaining health behavior in African countries, there is no reason to directly assume that sociocognitive models will be less suitable for understanding health behavior in African countries than in industrialized countries, apart from the fact that one could argue that the impact of social and environmental factors may be different and potentially greater in African countries. Studies are needed to assess this assumption and should analyze whether these factors, also outlined in ecological models (Sallis, Owen, & Fisher, 2008), may have a direct influence on behavior and/or are mediated via cognitions. If the former is true, this could explain why social cognitive factors may only moderately explain health behavior. Yet, another explanation is that previous studies did not thoroughly identify all salient beliefs that are relevant for understanding a particular behavior in a particular context. This implies a more thorough preparation using qualitative interviews in order to assess the most salient beliefs to be used in later quantitative research (Ajzen & Fishbein, 1980; Bartholomew, Parcel, Kok, Gottlieb, & Fernandez, 2011). Finally, the conditions of measurement are important. It may be that assessments in schools with classes of over 50 students with limited privacy have an impact on the quality, as well as generic educational levels (Wöβmann & West, 2006).

Strengths of the current study were the large sample size, its longitudinal design, the statistical technique used to assess motivational pathways, and the consistent operationalization of constructs between sites. Our study was subject to some limitations as well. First, measuring condom use among adolescents is difficult. Although anonymous self-report questionnaires were used, results could be biased due to socially desirable responses. The prevalence of condom use found in our study was, however, comparable to the prevalence reported by other studies (Reddy et al., 2010; UNAIDS, 2010). In addition, including the overall frequency and consistency of condom use, as well as current relationship status and the various pre-behaviors of condom use (i.e., buying, carrying and negotiating), is recommended for future research. Second, we did not measure the importance of attitude items to the participant or the motivation to comply with parents or friends. As a result, no multiplicative method (i.e., expectancy-value method) was applied in which attitudes are multiplied by their importance and subjective norms by the motivation to comply. Although some studies suggest that this approach has no benefits (Armitage, Conner, Loach, & Willets, 1999; Gagné & Godin, 2000; Trafimow & Finlay, 2002), Fishbein and Ajzen (2010) refuted these results and noted that the methodology used in most of these studies was flawed. Third, only one item was used to assess intentions to use a condom. The use of multiple questions would have increased the reliability of our results. Fourth, three different sites were compared based on measures at the individual level. No higher level variables, such as religion, school culture, school qualifications, or geographic location (urban versus rural), were included. It is likely that a large proportion of the unexplained variance in our study could be accounted for by site- or school-level variables. Fifth, future studies are recommended to assess potential gender differences in model fit and predictive value. The current study was unable to explore such differences due to the limited number of sexually active female participants.

### Conclusion

First, the results of this study imply that the TPB as a predictive model of condom use in sub-Saharan Africa is moderately successful. This suggests that selecting the TPB for the development and evaluation of condom use interventions in sub-Saharan Africa is justifiable. Therefore, other explanations, such as implementation issues or design issues, must be sought for the lack of increased effectiveness among theory-based interventions (Michielsen et al., 2012). Previous research has suggested that the following factors may need more attention in sub-Saharan programs: inclusion of moderators of effect, training of facilitators, methodological design, longer term follow-up measurements (> 1 year), involvement of stakeholders in the development of the program, and a dyadic approach that addresses gender inequity (Paul-Ebhohimhen, Poobalan, & van Teijlingen, 2008; Scott-Sheldon, Walstrom, Harrison, Kalichman, & Carey, 2013). Second, the contribution of attitudes, subjective norms, and self-efficacy seems to differ across regions within sub-Saharan Africa. Future research should focus on identifying those contextual factors that explain the differential predictive value of these constructs. Third, while attitudes were found to be the most important predictor of intentions in Europe and the U.S., most sub-Saharan studies, including the current study, showed that attitudes might have smaller effects on intention in sub-Saharan Africa. This implies that, instead of focusing solely on the individuals’ beliefs, structural approaches and an emphasis on coping with or changing societal norms must be included in future HIV-prevention programs. Future interventions that aim to promote condom use should use an integrative approach that takes into account both individual and contextual factors, as well as social and environmental differences (Gupta, Parkhurst, Ogden, Aggleton, & Mahal, 2008; Wegbreit, Bertozzi, DeMaria, & Padian, 2006).
